# Stromatolites and pulsed oxygenation events in the Mesoproterozoic Longjiayuan formation of western Henan: evidence for life-environment co-evolution

**DOI:** 10.1038/s41598-025-13303-w

**Published:** 2025-07-29

**Authors:** Qianqian Li, Deshun Zheng, Fengbo Sun

**Affiliations:** School of Resources and Environment, Henan Polytechnic University, Jiaozuo, 454003 China

**Keywords:** North China Craton, Mesoproterozoic, Stromatolites, Redox conditions, Ce/Ce* anomaly, Biogeochemistry, Ocean sciences

## Abstract

The oxygen level in the ocean and atmosphere played a crucial role in the development of Precambrian stromatolites. The Mesoproterozoic represents a critical interval in Earth’s history, characterized by persistently low atmospheric and oceanic oxygen levels under which stromatolites flourished. Stromatolites are particularly abundant in Member II of the Mesoproterozoic Longjiayuan formation in western Henan Province. To investigate the relationship between stromatolites and redox conditions, petrographic and geochemical analyses were conducted on siliceous-banded dolomites and stromatolitic dolomites from the Longjiayuan Formation. The results show that the macromorphology of stromatolites in Member II is classified into stratiform, undulatory, domical, conical, and columnar morphologies. Microscopically, the stromatolites exhibit alternating light and dark laminae, with occasional ooids and spherules. The Ce_SN_/Ce_SN_* values recorded in carbonate rocks from Member II show negative anomalies, reflecting oxidizing conditions influenced by environmental changes and microbial activity. These stromatolite records and oxygen fluctuations in the Mesoproterozoic shallow marine environment provide a valuable basis for studying the co-evolution of early life and the environment.

**Supplementary Information:**

The online version contains supplementary material available at 10.1038/s41598-025-13303-w.

## Introduction

Oxygen is a critical factor in the evolution of early life^1–3^. The Mesoproterozoic (1.8–0.8 Ga), often referred to as the “Boring Billion”, was originally characterized by stable δ¹³C records, and later recognized for persistent low oxygen levels and trace element scarcity, reflecting a prolonged geobiological stasis^4^. However, recent studies suggest that the oxygen content during this period was more dynamic than previously thought. In the North China Craton, three main perspectives have emerged: pulsed oxygenation episodes, continuous oxygenation, and spatio-temporal heterogeneity in the redox landscape, all indicating frequent oxygen fluctuations during the Mesoproterozoic. As evidenced by carbonates from the Wumishan Formation (1.52*–*1.47 Ga) in the northern NCC show higher I/(Ca + Mg) values and negative Ce anomalies, suggesting significant pulsed oxygenation episodes^5^. Another study of the Gaoyuzhuang Formation in Jixian County shows progressive ocean oxygenation from Member II to Member IV, with an estimated oxic seafloor area of up to 30% by the top of Member IV^6,7^. A study of the black shales from the Hongshuizhuang Formation (~ 1.46 Ga) in the northern NCC indicates persistent euxinic conditions in the northern basin, which gradually gave way to anoxic*-*suboxic conditions toward the south, reflecting a heterogeneous oxygen distribution^8^. Similarly, multiple geochemical indicators from the Mesoproterozoic (Lower Riphean) Arlan Member of the Kaltasy Formation (1414 ± 40 Ma, 1427 ± 43 Ma) suggest spatial redox heterogeneity in oxygen concentrations in deep ocean environments^9^. Further evidence supporting Mesoproterozoic oxygenation events comes from various global regions. For example, Planavsky et al., (2018) suggest that although the Mesoproterozoic ocean was primarily oxygen-poor or hypoxic, short-term oxygen fluctuations reflect a transitional state between the anoxic Archean and predominantly oxic Phanerozoic oceans^10,11^.

Researchers have proposed that the study of stromatolites can serve as an effective indicator of ancient marine environments^12^. Stromatolites are specialized biological sedimentary structures formed by the binding and trapping of calcareous sediments in atmospheric or marine settings, primarily driven by microorganisms such as cyanobacteria and other microbes. The formation of different microstructures in stromatolites is influenced by oxygen levels^13^. Bosak et al. (2009, 2010) proposed that the formation of fenestrae and bubble structures in Archean stromatolites was a result of oxygen production by photosynthetic microbial mats, which led to the entrapment of oxygen-rich bubbles within the microbial layers^14,15^. The microbialites that developed in the Jixian Group (1.6*–*1.4 Ga) located on the northern margin of the Mesoproterozoic NCC contain well-preserved microbial mats and cyanobacteria, and researchers have identified structures within the distribution patterns of these microbial mats that are associated with photosynthesis, such as alternating light-dark laminae and hourglass structures, which are linked to oxygen release^5^. Circular, concentric rosette structures formed by the stratification of organic matter are observed in the microscopic carbonate structures of columnar stromatolites in the Proterozoic McLeary Formation^16^. These geometries are consistent with the abiotic oxidation of biological carboxylic acids during diagenetic chemically oscillating reactions. Such reactions likely occur in early diagenetic environments when organic acids produced by the decomposition of organic matter are oxidized^17,18^. These chemically oscillating reactions may drive mineral precipitation in stromatolites, providing insights into the oxidation processes of deep stromatolitic environments^19^. Additionally, mineralogical observations of stromatolites from the Fengjiawan Formation in the Guandaokou Group, located on the southern margin of NCC, reveal the presence of pyrite, barite, and apatite as identified through SEM analysis. These minerals suggest microbially influenced precipitation processes and support the interpretation that cyanobacterial photosynthesis may have contributed to localized seawater oxidation^19^. Stromatolite morphologies have been shown to correspond to specific depositional environments, and thus serve as reliable paleoenvironmental indicators. For example, digitate stromatolite biostromes typically occur on intertidal flats along paleocoasts and are associated with phosphogenesis in shallow, low-energy environments, whereas columnar stromatolites form in deeper subtidal settings, where stable hydrodynamic conditions inhibit phosphogenesis^20^. Similar morphology–environment correlations have been reported in other Neoproterozoic and Precambrian successions. For instance, domical and undulatory forms are commonly observed in higher-energy or shallower zones, while conical and columnar morphologies are associated with lower intertidal to subtidal environments^21^. These relationships support using stromatolite morphological changes to infer shifts in depositional environments.

The strata of the Longjiayuan formation within the Guandaokou Group of the Mesoproterozoic in the western Henan region are well-preserved and exhibit continuous exposure, making it an ideal location for studying the marine environment of the Mesoproterozoic era. While numerous scholars have made significant contributions to understanding the stratigraphy of the southern NCC, research connecting stromatolites to the redox conditions of this period remains limited. This study focuses on analyzing the lithological and geochemical characteristics of siliceous-banded dolomites and stromatolites preserved in the Longjiayuan Formation section in the southern NCC. The aim is to reconstruct the sedimentary environment of the Longjiayuan formation and investigate the growth patterns of stromatolites during the oxygenation events of this period. Additionally, the study explores the relationship between redox conditions and microbial activity from multiple perspectives.

## Geological setting

**Fig. 1:**
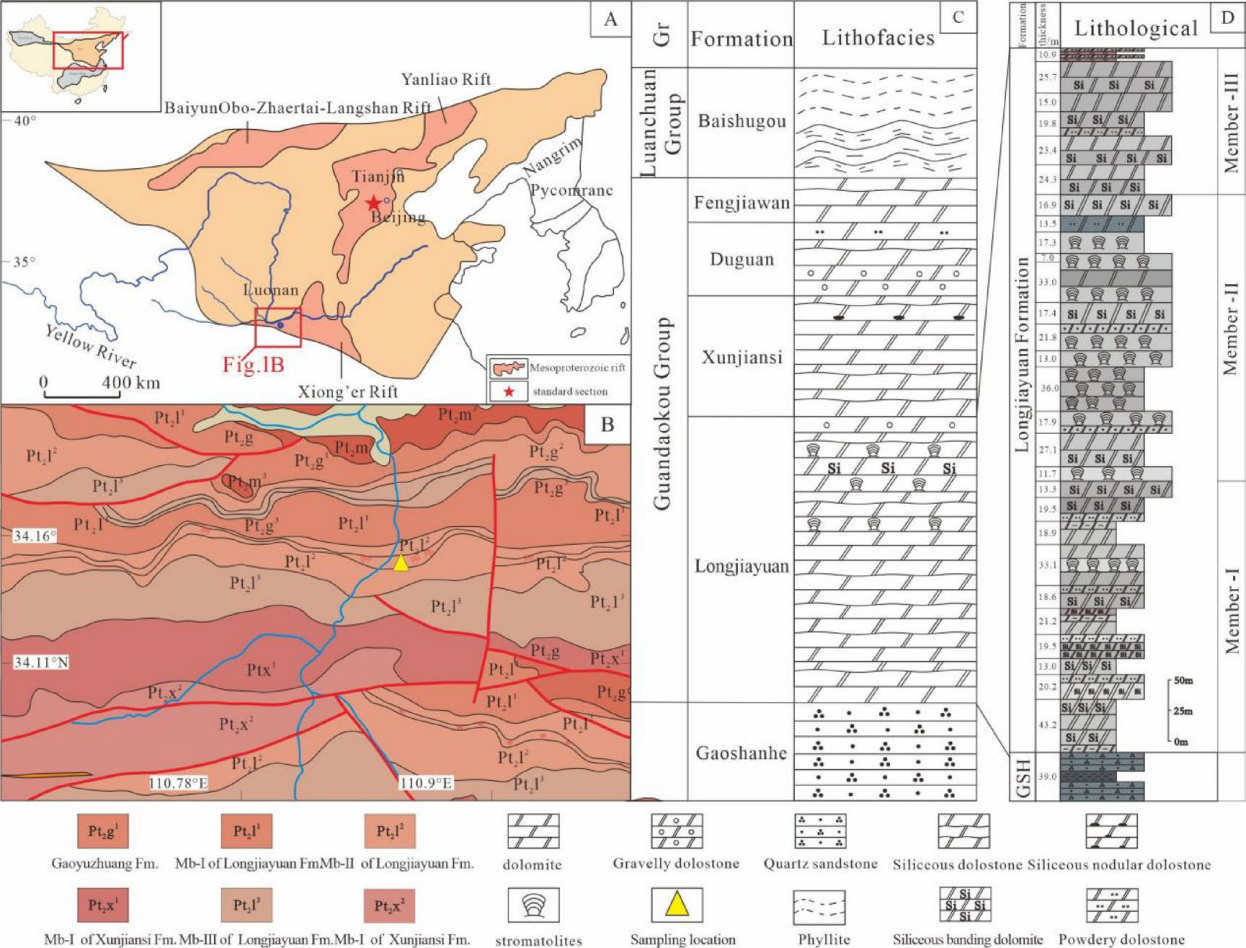
Geological setting of the study area. (**A**) Precambrian geological map of the North China Craton (modified after^25^. (**B**) Geological map and cross-section of the southern margin of the NCC, showing the location of the Guandaokou Group carbonates. The base map was geolocated using the GeoCloud App (China Geological Survey, http://www.geocloud.cgs.gov.cn), and the figure was finalized using CorelDRAW Graphics Suite 2020 (https://www.coreldraw.com). (**C**) Lithologic column diagram of Guandaokou Group in western Henan Province. (**D**) Lithologic column diagram of Longjiayuan Formation.

Following the formation of the basement of the NCC, the rift system began to develop and evolve, leading to the formation of the Xiong’er Rift System, the Yanliao Rift System, and the Zhaertai-Baiyun Obo Rift System (Fig. 1A). Subsequently, a widespread and continuous sequence of clastic and carbonate rocks was deposited over the crystalline basement^22^. In the southern NCC, these strata are predominantly found in western Henan Province and are divided into three main stratigraphic regions: the Songshan-Jishan region, the Mianchi-Queshan region, and the Lushi-Luachuan region. The Lushi-Luanchuan stratigraphic region is widely distributed across Lushi, Luoning, Luanchuan, and Fangcheng in Henan Province, extending westward into Luannan in Shan’xi Province^23^. The stratigraphic sub-unit contains a sequential deposition of the Mesoproterozoic Xiong’er Group, Gaoshanhe Group, and Guandaokou Group (which includes the Longjiayuan, Xunjiansi, Duguan, and Fengjiawan formations), followed by the Baishugou Formation (Fig. 1C). The Guandaokou Group is characterized by dolomitic carbonates with abundant stromatolites, indicating a carbonate platform sedimentary environment (Fig. 1D). Zhang et al., (2019) conducted SHRIMP zircon U-Pb isotopic geochronology on tuffaceous rocks from the lower and basal parts of the Longjiayuan formation in the Jiuligou-Sanchuan section, Luanchuan, Henan Province, determining an age of approximately 1.59–1.54 Ga^24^.

In this study, field reconnaissance, geological section measurement, and sampling were conducted on the siliceous-banded dolomite and stromatolitic dolomite of the Longjiayuan Formation. The Longjiayuan formation is located south of Fudi Village, Sucun Township, Lingbao City, Henan Province, with geographic coordinates of 34°19’02” N and 110°57’32” E (Fig. 1B). The section is well-exposed and consists of a carbonate rock sequence primarily composed of dolomite.It contains numerous siliceous bands, with abundant stromatolites. The bottom of the Longjiayuan formation is characterized by purplish-red quartz sandstone, which is unconformably in contact with the underlying Gaoshanhe group. The top is defined by interbedded strata of siliceous striped dolomite and thick-bedded dolomite, which are conformably in contact with the overlying Xunjiansi Formation. The formation has a total thickness of 611 m.

**Fig. 2:**
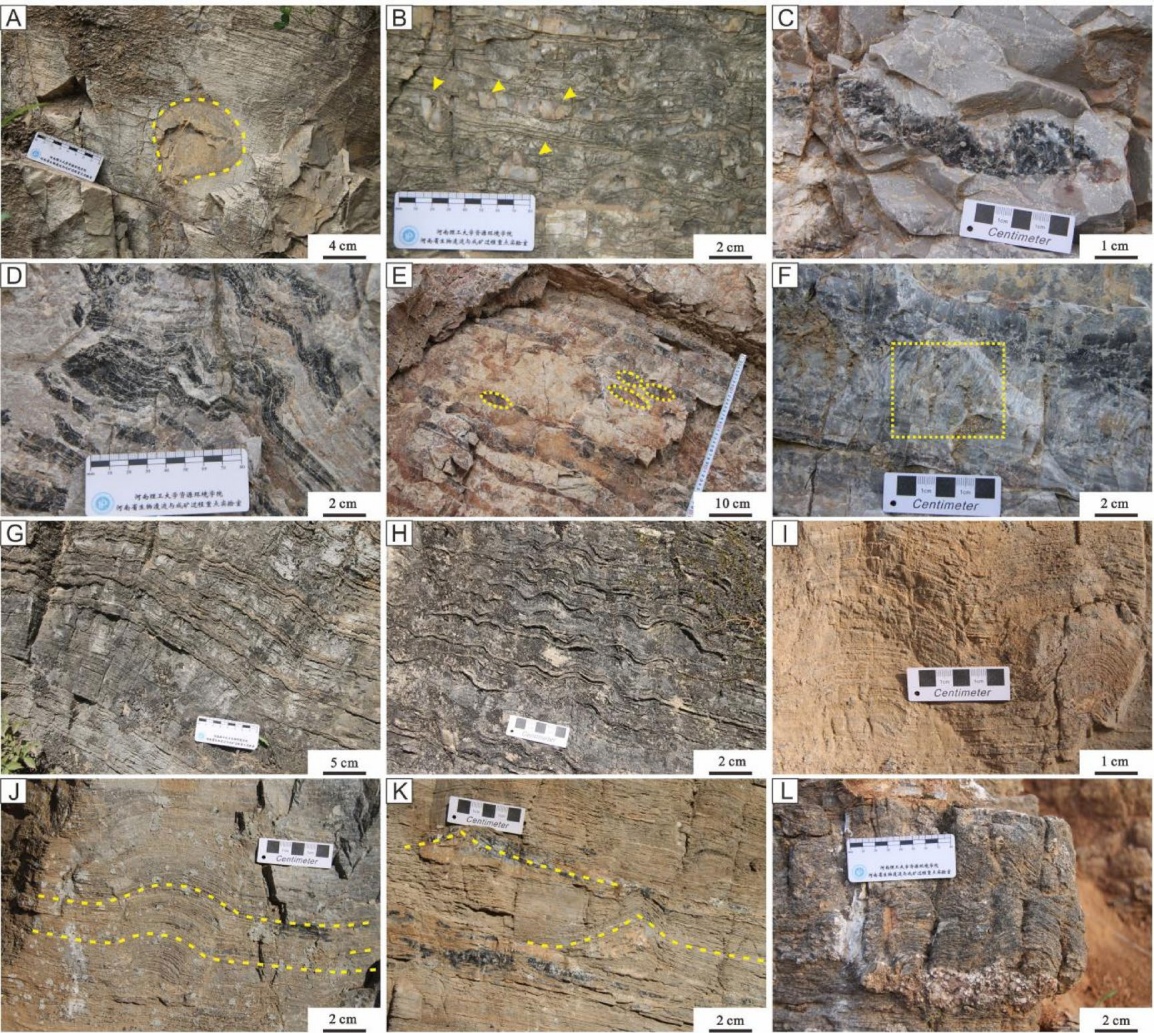
Field photographs showing representative lithologies of siliceous-banded dolomites and stromatolitic dolomites from the Longjiayuan formation in the southern NCC. (**A**,** B**) siliceous nodules. (**C**, **D**) siliceous bands. (**E**) siliceous nodules. (**F**) laminar structure. (**G**) stratiform stromatolite. (H) undulatory stromatolite. (**I**,** J**) domical stromatolite. (**K**) conical stromatolite. (**L**) columnar stromatolite.

The Longjiayuan formation can be broadly divided into three members based on lithological variations^26^. Member I is characterized by light gray and gray siliceous-banded dolomite and fine-crystalline dolomite, with the presence of siliceous nodules (Fig. 2A-E). Member II is dominated by light gray to gray stromatolitic dolomite and fine-crystalline dolomite, exhibiting diverse stromatolite morphologies such as stratiform, undulatory, domical, columnar, and conical forms (Fig. 2G-L). Member III primarily consists of light gray fine-crystalline dolomite, with a minor occurrence of stromatolites.

## Materials and methods

A total of 56 samples of siliceous strip dolomite and stromatolitic dolomite were collected. Fresh, non-weathered samples were selected for major and trace element analysis. Field observations were conducted macroscopically, while microscopic thin-section analysis was performed at the State Key Laboratory of Biological Relics and Metallogenic Processes, Henan Polytechnic University. A Carl Zeiss Axioskop 40 polarizing microscope was used for microscopic observations.

Whole-rock major and trace element analyses were performed by Wuhan Sample Solution Analytical Technology Co., Ltd. The sample pretreatment for whole-rock major element analysis was conducted using a fusion method to prepare glass disks. Analysis of the fused lithium borate glass disks was performed using X-ray fluorescence spectroscopy (XRF, ZSX Primus II) for carbonates. A standard curve was established using the national rock standard material series GBW07101-14, and the α correction coefficient method was applied. The analytical precision is better than 5%. For trace elements (including REY) analyses, ~ 50 g powdered sample were weighed and placed in a Teflon bomb, to which 1 ml of high-purity HNO₃ and 1 ml of HF were sequentially added. The Teflon bomb was sealed and heated for more than 24 h. After cooling, the sample was evaporated to dryness, followed by the addition of 1 ml of HNO₃ and further evaporation to dryness. Subsequently, 1 ml of high-purity HNO₃, 1 ml of MQ water, and 1 ml of internal standard solution (1 ppm In) were added. The Teflon bomb was resealed and heated for more than 12 h. The final solution was transferred to a polyethylene bottle and diluted to 100 ml with 2% HNO₃ for Agilent 7700e ICP-MS analysis. The measurement precision is better than 5%.

Carbon and oxygen isotopes analyses were conducted at ALS Chemex(Guangzhou) Co., Ltd. using the inorganic phosphoric acid method to release CO₂. The released CO₂ was introduced into a Thermo-Finnigan GasBench system connected to a MAT DeltaPlus isotope ratio mass spectrometer (CF-IRMS) for carbon and oxygen isotope measurement. Analytical precision for carbon and oxygen isotopes is better than 0.2‰.

## Results

### Petrographic characteristics

Member I of the Mesoproterozoic Longjiayuan formation is dominated by siliceous-banded dolomite. The siliceous material is primarily composed of chalcedony and microcrystalline quartz. The formation of siliceous rocks requires sufficient silica sources and specific geochemical conditions. Its simple composition and stable structure give it strong resistance to late-stage alteration and minimal influence from late diagenesis, allowing it to better preserve the geological information from its time of formation. The siliceous nodules in the Member I are elliptical in shape, characterized by a distinct boundary that clearly separates the nodules from the surrounding rock (Fig. 2A). In addition, dense siliceous laminae are interbedded with siliceous masses, where the intact laminae are disrupted by the irregular distribution of these masses (Fig. 2B). The shape of the black siliceous bands within the dolomite is notably irregular, further illustrating the complex internal structure of the formation (Fig. 2C, D). Most of the siliceous bands are oval-shaped and aligned parallel to the bedding planes (Fig. 2E). In addition, needle-like or spinose structures are observed within the dolomite matrix, exhibiting tapered, conical morphologies (Fig. 2F). These spiniform structures are believed to form through the accumulation of microbial mat fragments after the mats have undergone consolidation or semi-consolidation. Due to the large pores and open spaces within these structures, they are particularly prone to processes such as silicification or dolomitization.

The lithology of the Member II primarily consists of light gray, gray stromatolitic dolomite, and fine-grained dolomite, often associated with siliceous bands and masses. Based on their stratified morphology, the stromatolites are categorized into stratiform, undulatory, domical, conical, and columnar forms. The stratiform stromatolites exhibit nearly horizontal, continuous laminae with minimal undulation. The distance between the layers is uniform, and each individual layer is thin, measuring approximately 0.5 to 1 mm, forming a compact structure. The stromatolites have a general gray-black appearance, with alternating light and dark-grained layers, and the surrounding rock consists of fine-grained dolomite (Fig. 2G). The laminae of the undulating stromatolites are wavy, with regular undulations. The diameter of individual undulations is approximately 2 cm, and the size of the wave crests and troughs is uniform. The thickness of the laminae gradually decreases from the bottom to the top, while the curvature of the undulatorys progressively increases (Fig. 2H). Surface weathering has caused the undulatorys to protrude from the rock surface. The domical stromatolites exhibit regular convex-upward laminae, with a thickness of approximately 2 mm that becomes more uniform from bottom to top. The curvature of stromatolitic laminae is regular, with convex arcs uniformly oriented in the same direction (Fig. 2I, J). The conical stromatolites exhibit symmetrical, inclined laminae gradually converging toward a central apex. The laminae are thin and densely packed. Conical stromatolites are developed within intervals dominated by thick stratiform stromatolites. The weathered surface appears earth-yellow, with siliceous bands and masses, and occasional development of concentric or elliptical oncoids (Fig. 2K). The columnar stromatolites are cylindrical, approximately 5 cm in diameter. The thickness of the laminae is approximately 1 mm, and the laminae are densely packed.This may be due to the high organic matter content, with the laminations predominantly consisting of dark-colored layers (Fig. 2L). Linked columnar stromatolites exhibit diverse morphologies. In some cases, two independent stromatolite columns are merged and connected, while in other instances, the columns may separate and then reconnect, forming an overlapping structure. The lithology of Member III of the Longjiayuan formation is dominated by fine-crystalline dolomite, with occasional occurrences of stratiform stromatolites. Toward the top of the succession, thick-bedded gray dolomicrite with upward-decreasing grain size relative to the underlying strata is developed.

### Microscopic characteristics

**Fig. 3:**
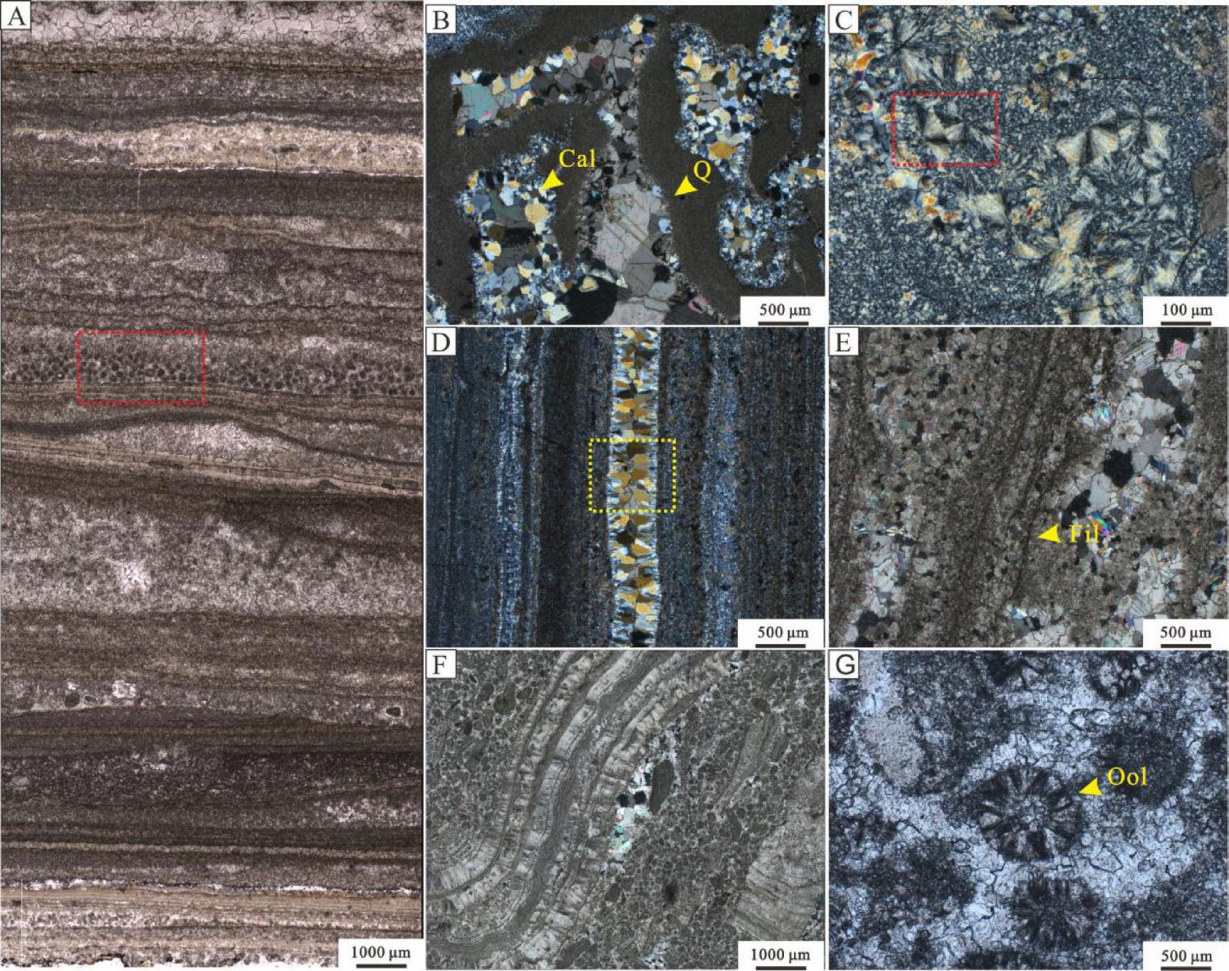
Optical petrography of siliceous-banded dolomites and stromatolitic dolomites from the Longjiayuan Formation, southern NCC. (**A**) Micro-laminations of stromatolite showing microspherules (red box), under plane-polarized light (PPL). (**B**) Microstructure of siliceous-banded dolomite, illustrating calcite and quartz replacement, under crossed-polarized light (XPL). (**C**) Siliceous-banded dolomite with radiating chalcedony (red box) (XPL). (**D**) Siliceous-banded dolomite showing quartz (yellow box) (XPL). (**E**) Stromatolite lamina with filaments (yellow arrow) (XPL). (**F**) Stromatolite lamina showing limited ooids and calcite cement at the edge of the lamination (XPL). (**G**) Radial ooids (yellow arrow) (PPL). (CAL = calcite, OOL = ooids, Q = quartz).

Alternating light and dark laminae are visible on the stromatolite microlaminae (Fig. 3A, Fig. S1), with the darker, microbe rich laminae in the lower part varying in thickness and containing microfilamentous structures that are unevenly distributed, while synsedimentary cement fills the fenestrae and intergranular porosity; A spherulite layer develops in the middle part, where the spherulites are predominantly micritic solid spheres or ellipsoids, with small individual aggregates (< 200 μm) that typically form in clusters. The radial chalcedony in the siliceous band (Fig. 3C) is primarily composed of quartz and quartz cement. The quartz band in the siliceous layer is clearly distinguishable from the surrounding microcrystalline quartz (Fig. 3D). Calcite occurs between the laminae in stromatolites (Fig. 3E). In stromatolites (Fig. 3F), the laminae vary in thickness, and spherules of various sizes are densely distributed around the layers, with calcite development in the middle. Three types of microstructures were observed in the laminae: fan-shaped layers, micritic layers, and fan-micrite mixed layers. The micritic layer contains clastic quartz particles bound to the structure, while the fan-shaped layer exhibits typical full extinction indicative of non-biological precipitation. Concentric-radial ooids (Fig. S2), approximately 300 μm in size, were observed in stromatolites (Fig. 3G). The outer rim of the ooids is smooth and circular, with radial striations composed of radiating fibrous organic material.

### Geochemistry

#### Major elements composition

The major element compositions of siliceous-banded dolomites and stromatolites from the Longjiayuan formation were analyzed. The SiO₂ content in siliceous striated dolomites from Member I of the Longjiayuan formation ranges from 0.75 to 88.46 wt%, with an average of 35.3 wt%. In Members II to III, the SiO₂ content decreases significantly, ranging from 0.02 to 22.6 wt%, with an average of 3.52 wt%. The average contents of Al₂O₃, K₂O, TFe₂O₃, TiO₂, and MnO are very low, measured at 0.09 wt%, 0.03 wt%, 0.18 wt%, 0.01 wt%, and 0.15 wt%, respectively. Stromatolites exhibit CaO and MgO contents ranging from 9.81 to 30.66 wt% and 10.87 to 21.88 wt%, with averages of 24.9 wt% and 17.86 wt%.

#### Rare earth elements and yttrium (REY)

**Fig. 4:**
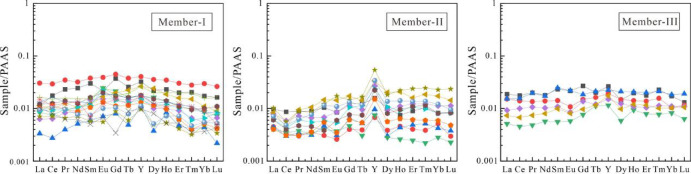
REE distribution pattern diagram. **A**−**C** show the normalized distribution patterns of siliceous-banded dolomites and stromatolites from Members I, II, and III of the Longjiayuan Formation, respectively. PAAS normalized values are derived from^27^.

The total rare earth element (REE) content of samples from the Longjiayuan formation ranges from 0.03 to 1 ppm, with an average value of 0.11 ppm. The LREE/HREE ratio varies from 1.27 to 2.9 ppm (avg. 1.88). Elemental concentrations were normalized to Post-Archean Australian Shale (PAAS)^27,28^ denoted as SN in the following discussion. After PAAS normalization of the dolomite and stromatolite samples from the Longjiayuan Formation, the REY distribution patterns are illustrated in Fig. 4A–C. Based on established methods, the following equations were applied to calculate REY anomalies in the dolomite and stromatolite samples from the Longjiayuan formation:

Ce/Ce* = 2Ce_SN_/(La_SN_ + Pr_SN_)^28^.

Eu/Eu* = 2Eu_SN_/(Sm_SN_ + Gd_SN_)^29^.

La/La* = La_SN_/((Pr_SN_ + Nd_SN_)/2)^29,30^.

Gd/Gd* = Gd_SN_/((Sm_SN_ + Tb_SN_)/2)^29^.

The results show that the Ce_SN_/Ce_SN_* values of the Longjiayuan formation range from 0.32 to 1.66 ppm(avg. 0.89), indicating variable cerium anomalies from negative to slightly positive. The Eu/Eu* values range from 0.55 to 1.76 ppm (avg. 0.98), indicating no significant Eu anomalies. The La/La* values range from 0.47 to 1.67 ppm (avg. 1.01), showing no to slightly positive La anomalies. In contrast, the Gd/Gd* values vary from 0.49 to 1.53 ppm (avg. 1.16), suggesting a slight enrichment in middle REEs.

#### Carbon and oxygen isotopes

The δ¹³C values of the Longjiayuan formation range from − 1.42‰ to 1.28‰ (avg. −0.14‰), reflecting generally negative carbon isotope compositions with occasional positive excursions. The δ¹⁸O values vary from − 7.78‰ to − 3.99‰ (avg. −6.09‰), showing considerable fluctuation (Fig. 7).

## Discussions

### Evaluation of diagenetic alteration and detrital contamination

**Fig. 5:**
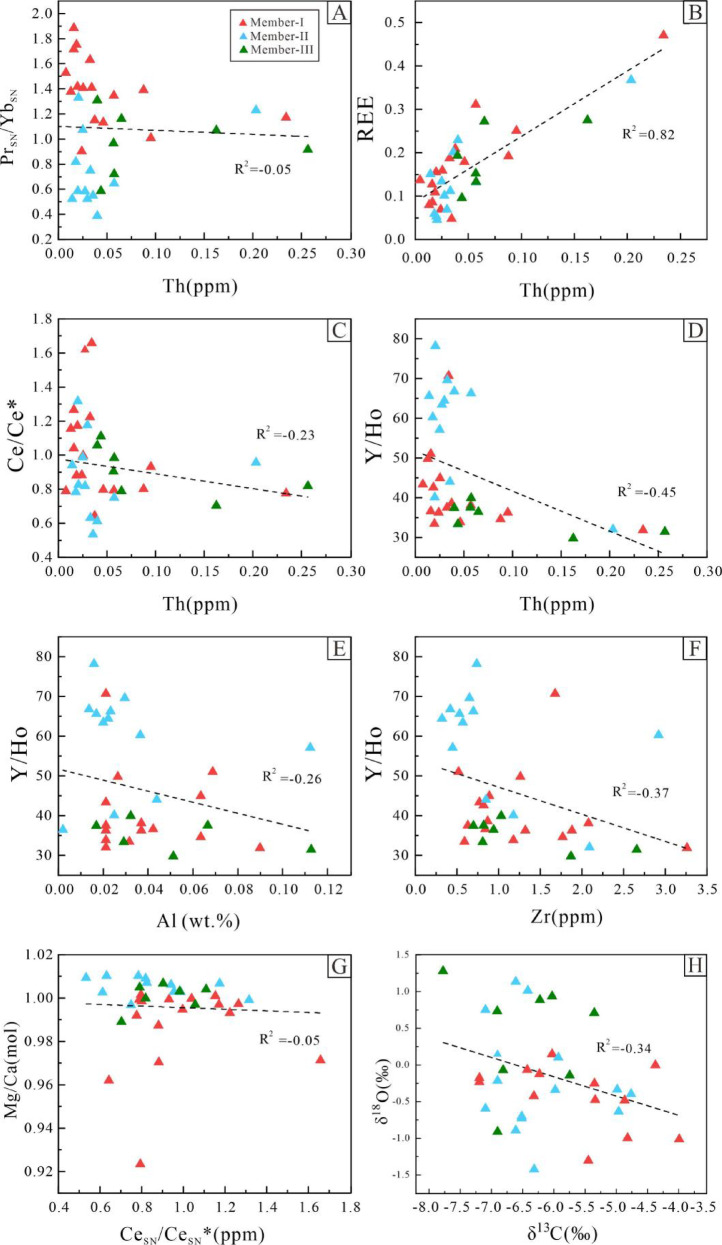
Geochemical Element Analysis. **A**−**H**: Covariance plots of Th vs. Pr_SN_/Yb_SN_*, Th vs. REE, Th vs. Ce_SN_/Ce_SN_*, Th vs. Y/Ho, Th vs. Al(wt%), Th vs. Zr, Ce/Ce* vs. Mg/Ca, and δ¹³C (‰) vs. δ¹⁸O (‰).

Two major factors affecting the reliability of carbonate REYs data are diagenetic alteration and terrigenous clastic input^31,32^. While other REEs in carbonate rocks are generally less sensitive to diagenesis, diagenetic processes can still significantly modify the Ce_SN_/Ce_SN_* ratios in marine carbonates^33^. Mg/Ca ratios provide important information for evaluating diagenetic alteration in carbonates. During dolomitization, Mg^2+^ replaces Ca^2+^, leading to an increase in Mg/Ca ratios. If these ratios show no significant increase, it suggests that dolomitization has not notably altered the rocks^34^. In Fig. 5G, the absence of a significant increase in Mg/Ca ratios further reinforces the conclusion of minimal diagenetic alteration. Thorium (Th) is a proxy for terrigenous input, as the REYs content in detrital minerals is much higher than in seawater. Even small amounts of terrigenous clastic input (approximately 1%) can significantly alter the REYs signal of carbonate rocks^31^. Therefore, Th content is commonly used as an indicator of terrigenous input in correlation analyses^32^. In the Longjiayuan formation samples, no significant correlations were observed between Th and REY parameters such as Pr_SN_/Yb_SN_*, Ce_SN_/Ce_SN_*, and Y/Ho (Fig. 5A, C, D), indicating that these indices were not notably influenced by terrigenous clastic input. Although Th exhibits a positive correlation with REE (Fig. 5B), suggesting that the total REE concentrations may be partially affected by detrital components, additional immobile elements such as Al and Zr were incorporated to more comprehensively assess detrital contamination. Zr is primarily hosted in zircon, a stable heavy mineral commonly found in clastic sediments, and serves as a reliable proxy for terrestrial input. Moderate negative correlations between Y/Ho and both Al (Fig. 5E) and Zr (Fig. 5F) suggest that the extent of detrital contamination is generally limited, and that most samples retain REY patterns indicative of deposition from oxygenated seawater. Furthermore, the covariation between La/La* and Gd/Gd* (Fig. S3) shows that most samples exhibit slight positive anomalies. These anomalies may reflect the adsorption and enrichment of REEs by Fe-Mn oxyhydroxide particles and clay minerals during sedimentation, suggesting that the REY distributions were primarily governed by seawater-derived processes^30^. Additionally, the correlation between δ^13^C and δ^18^O values in the carbonate rock samples is weak (R² = 0.12, Fig. 5H), suggesting that the δ^13^C and δ^18^O values in post-depositional samples are minimally influenced by diagenesis and can accurately reflect the isotopic composition of ancient seawater during the initial deposition of the Longjiayuan formation^35^.

Due to their similar ionic radii, Y^3+^ and Ho^3+^ are considered to exhibit similar geochemical behavior in marine carbonate depositional environments. The Y/Ho ratio in freshwater, such as rivers, closely matches the average value found in post-Archean shale. However, due to differences in surface complexation capacity, the precipitation rate of Ho from seawater is approximately twice that of Y. As a result, Y/Ho ratios are often used as indicators to distinguish between marine and terrestrial sediments. Y/Ho ratios in Archean and post-Archean microbial carbonates deposited under marine conditions typically exceed 40^33,36–38^. The Y/Ho ratios of dolomites and stromatolites from the Longjiayuan formation range from 29.73 to 78.16, with an average of 49.06, reflecting the REE characteristics that record seawater signals. After normalizing the dolomite and stromatolite samples to PAAS, the REY partitioning curve is shown in Fig. 4A-C. The Members I and III of the Longjiayuan formation exhibit no significant anomalies and display flat REY patterns. In contrast, the REY pattern of Member II shows typical light rare earth element (LREE) depletion relative to heavy rare earth elements (HREE), along with a weak negative Ce anomaly and a positive Y anomaly. This pattern closely resembles that of modern oxygenated seawater, indicating that the REY signature likely reflects deposition under oxic marine conditions.

In summary, the REE geochemical composition of the Longjiayuan formation samples is minimally altered by diagenesis and primarily reflects the influence of seawater, with little impact from terrigenous detritus. Therefore, it can be concluded that the rare earth characteristics of the siliceous-banded dolomites and stromatolite samples in the study area predominantly reflect their seawater sources.

### Sedimentary environment of the Longjiayuan formation

Siliceous bands dolostones in Member I of the Mesoproterozoic Longjiayuan formation in western Henan Province exhibit a variety of morphologies, including laminated-banded, deformed laminar, nodular-clump, and chrysanthemum-like types. These morphologies respectively reflect environmental processes such as alternating precipitation, bioturbation, localized siliceous enrichment, and rapid deposition^39^. Some studies have pointed out that the formation of siliceous bands was closely linked to environmental microbial activity during this period. For instance, during intervals of intensified microbial activity, organic matter accumulated on the seafloor, causing a decrease in pH near the sediment–water interface. This reduction in pH led to a decline in the solubility of silica, and upon reaching supersaturation, significant amounts of silica precipitated to form siliceous bands. The periodicity of microbial activity further resulted in banded rhythmic layers characterized by interbedded dolomite and siliceous deposits^40,41^. The major element composition of dolostones from Member I shows high SiO₂ contents, and replacement textures between quartz and calcite are observed (Fig. 3B), suggesting that the siliceous components may have originated from secondary precipitation or later diagenetic replacement. Stromatolites are sparsely developed and primarily formed as stratiform morphologies, indicating deposition under low-energy hydrodynamic conditions. These observations collectively suggest that Member I was deposited in a shallow marine environment, likely within the intertidal to supratidal zones. Stromatolites in this interval are sparsely developed and mainly exhibit flat-laminated morphologies, indicative of low-energy hydrodynamic conditions. Taken together, these observations suggest that this part of the Longjiayuan formation was deposited in a shallow marine intertidal to supratidal environment.

The Member II of the Longjiayuan formation is characterized by the abundant development of stromatolites. The sedimentary environment of stromatolites is classified based on their macroscopic morphology and microscopic characteristics. The lithology of the lower part of Member II consists mainly of gray and black thick-bedded, fine-grained dolomite. Stratiform stromatolites are horizontally developed and characterized by straight, laterally continuous, and densely laminated structures with substantial sedimentary thickness. Figure 3A (red box) shows dense laminae composed of spheroids, typically about 5 μm in diameter, appearing either as individual globular microfossils or small aggregate communities. This morphology is similar to the abundant globular features found in stromatolitic laminae of the Wumishan Formation on the northern margin of NCC^42^. Similar spheroidal structures have been interpreted as compact micritic bodies within microbial mats, and are commonly associated with coccoid cyanobacteria^42^. These features may reflect microbially induced carbonate precipitation (MICP), including possible mineralization of cyanobacterial colonies or carbonate nucleation mediated by extracellular polymeric substances (EPS) during early diagenesis. The central part of Member II is primarily composed of gray or gray-black thick-bedded, medium-crystalline stromatolitic dolomite. domicals and undulating stromatolites are well-developed, with the surface laminae of the undulating stromatolites showing good continuity and pronounced undulation. This reflects that the water environment was stable during deposition, with uniform precipitation rates across different sedimentary layers, suggesting a subtidal environment. Domicals, conicals, and columnar stromatolites typically develop in intertidal or subtidal zones with high water energy^21^. Compared todomicals, columnar stromatolites require stronger water energy. The presence of domicals, conicals, and columnar stromatolites indicates that hydrodynamic conditions were gradually intensifying, placing the strata in a intertidal zone environment^43^. Based on the variations in stromatolite characteristics, the middle part of Member II represents a transitional sedimentary environment between the intertidal and subtidal zones. In the upper part of Member II, well-developeddomicals, columnar, and conical-shaped stromatolites are observed. The columnar stromatolites contain more dark laminae, with irregular microbial laminae resembling flocculent and misty structures. Filamentous bodies indicate organic-rich laminae, composed of intertwined filaments. Concentric-radial oolitic particles (Fig. 3G, Fig. S2), consisting of micrite and sparry calcite, are observed microscopically in the conical stromatolites. Subsequently, Stratiform stromatolites with minimal laminae undulation developed in low-energy water environments, reflecting deposition of shallow marine stromatolites in a stable setting without significant hydrodynamic disturbances, characteristic of an subtidal environment. Overall, the upper part of Member II represents a transitional deposit between the subtidal and intertidal zones. Member III of the Longjiayuan formation is characterized by grey fine-crystalline dolostones and stratiform stromatolites. In the lower part of Member III, siliceous bands become progressively thicker, reflecting changes in silica supply to the depositional environment. In the upper part, the stratiform stromatolites exhibit compact and regular laminations, indicating calm water conditions with minimal disturbance. These features collectively suggest a low-energy subtidal setting during this depositional stage.

**Fig. 6:**
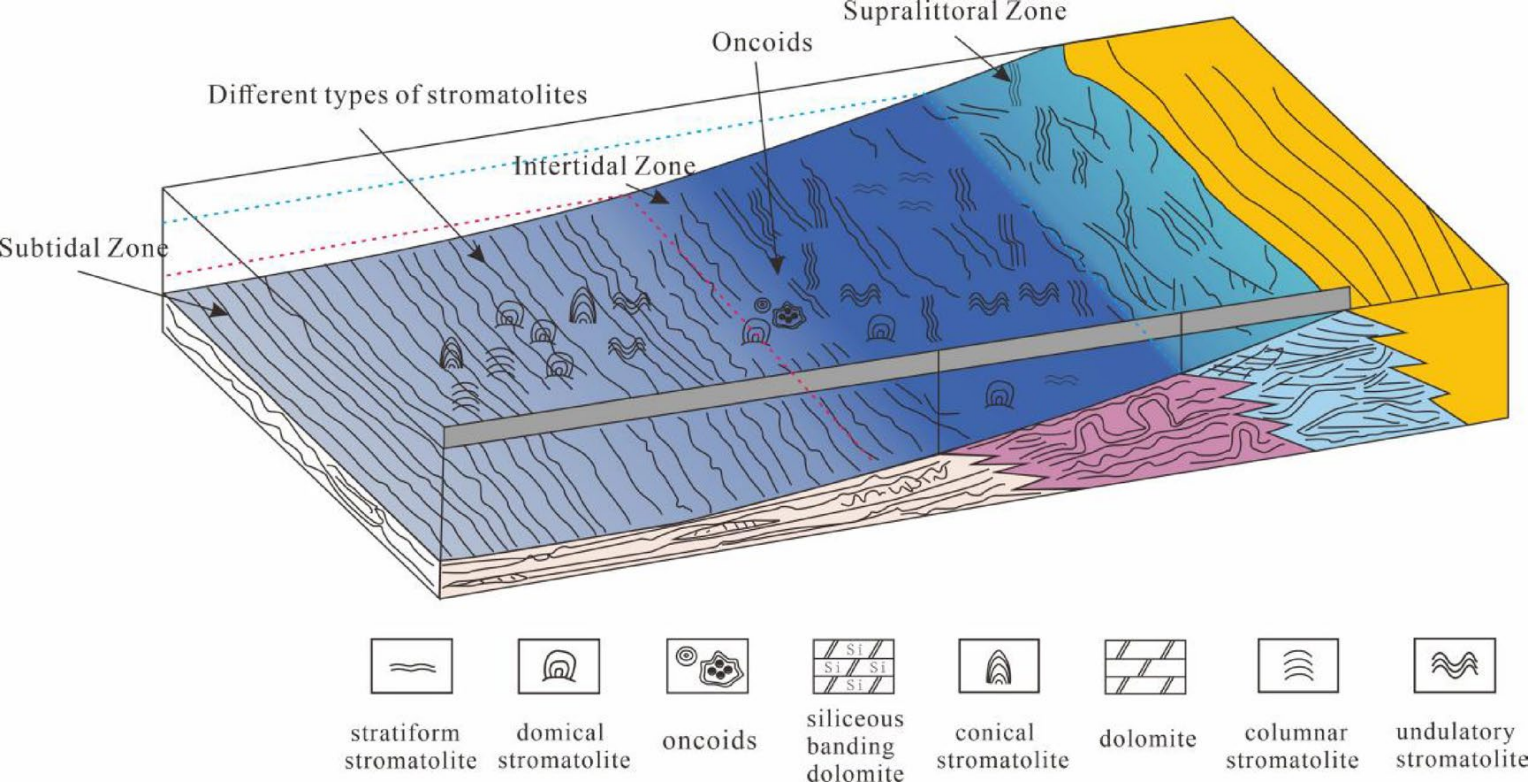
Schematic sedimentary development model for the Longjiayuan formation stromatolites (modified after^44^).

During the early stages of sedimentation in western Henan, the seawater was shallow (Fig. 6). The overall sedimentary characteristics of the Longjiayuan formation indicate a transition from intertidal-supratidal settings to subtidal environments, with transitional features preserved in Member II.

### Redox conditions of the Longjiayuan formation

To accurately reflect the changes in redox conditions during the deposition of siliceous-banded dolomites and stromatolites in the Longjiayuan formation, REE Ce_SN_/Ce_SN_* values were used to comprehensively assess the redox conditions of the water. Cerium is unique among the REEs as it can exist in both + 3 and + 4 oxidation states. Under oxic conditions, Ce(III) is partially oxidized to Ce(IV), which is preferentially removed from seawater by settling Mn oxides, resulting in a depletion of Ce relative to other trivalent REEs in the water column^45–47^.

**Fig. 7:**
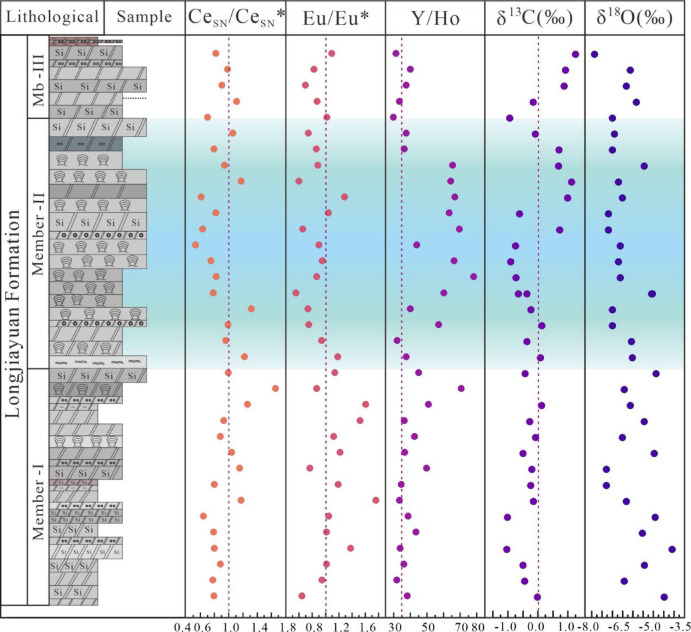
The geochemical elements variation diagram of the Longjiayuan formation.

In the geochemical element variation map (Fig. 7), within Member I (FD-1 to FD-38), Ce_SN_/Ce_SN_* values varied significantly (Ce_SN_/Ce_SN_* = 0.32–1.66), reflecting fluctuations in the redox conditions of the sedimentary environment at different horizons. In Member II (FD-40 to FD-89), most Ce_SN_/Ce_SN_* values were concentrated between 0.43 and 1.32, with values in this range being low. Notably, in the stromatolite-bearing horizon (FD-57, Ce_SN_/Ce_SN_* = 0.43), the oxygen concentration in the environment was elevated, possibly due to more open water conditions and enhanced oxygen exchange. However, Some samples exhibited higher Ce_SN_/Ce_SN_* values (FD-48, Ce_SN_/Ce_SN_* = 1.32), suggesting areas with limited oxygen supply, where the sedimentary environmen texhibited certain restrictive characteristics. The Ce_SN_/Ce_SN_* values in Member II exhibit spatiotemporal heterogeneity in the redox landscape. In Member III (FD-90 to FD-101), Ce_SN_/Ce_SN_* values were generally close 1 (avg. 0.97), suggesting near-neutral Ce anomalies and indicating a weakly oxidizing to suboxic environment.

In summary, the Ce_SN_/Ce_SN_* values from the Longjiayuan formation demonstrate significant fluctuations in the redox conditions during deposition. The periodic variations in water oxygen concentration were closely linked to changes in water column depth, environmental shifts and microbial activity. Thus, the changes in Ce_SN_/Ce_SN_* values not only provide direct evidence of redox conditions but also reflect the dynamic shifts in the sedimentary environment.

### Stromatolite growth and the diversity of mesoproterozoic redox conditions

Siliceous-banded dolostones and siliceous nodules are widely developed in Member I of the Longjiayuan formation, with limited occurrences of stromatolites. Slightly positive Ce anomalies suggest deposition under weakly reducing seawater conditions. Slight positive Eu anomalies observed in some samples of siliceous-banded dolostones (Fig. 7) may indicate episodic hydrothermal input^48,49^ which could have provided dissolved silica and further promoted the formation of siliceous bands. Similar models of hydrothermal silica deposition in shallow platform settings have been recognized in the Mesoproterozoic Wumishan formation^50^.

**Fig. 8:**
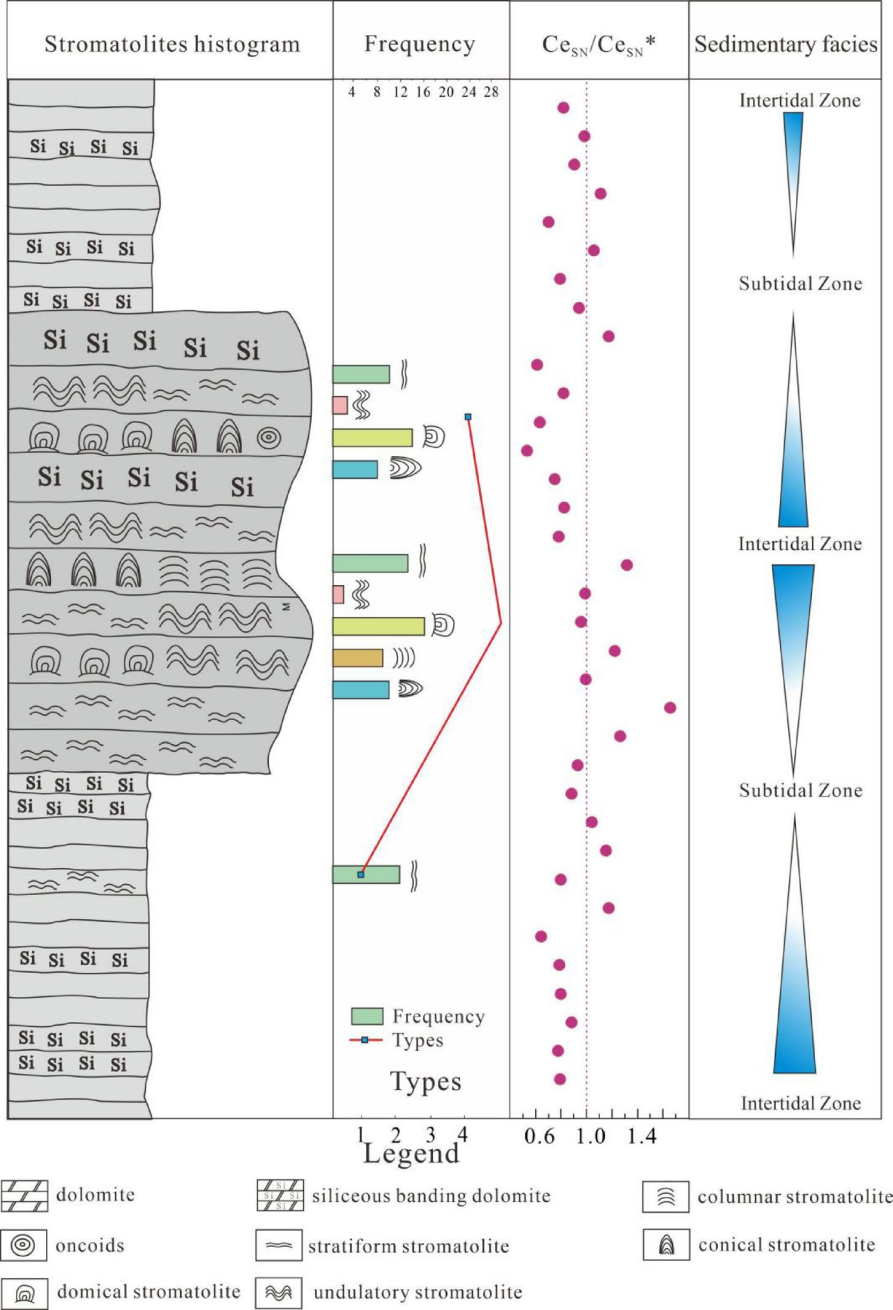
Stromatolite distribution, Ce anomaly (Ce_SN_/Ce_SN_*), and facies variation across the Longjiayuan formation.

Although changes in water depth may influence redox conditions by shifting the position of the redoxcline, geochemical evidence presented in this study suggests that the observed redox enhancement in Member II is more plausibly attributed to the mixing of terrigenous fluids with oxygenated open-marine waters^51,52^. The co-occurrence of negative Ce anomalies and elevated Y/Ho ratios indicates a substantial influx of external seawater during this period, which likely increased oxygen availability in the depositional environment. This process may have been driven by a combination of sea-level rise and reduced basin restriction, enhancing connectivity with the open ocean^53^. In addition, photosynthetic activity of stromatolites would have contributed to local oxygen production at the sediment-water interface. Therefore, the redox signals preserved in Member II were likely shaped by both enhanced open-ocean water input and localized photosynthetic oxygen production, rather than being governed solely by variations in water depth. At the base of Member III, Ce anomalies converge toward near-neutral values, indicating a rapid attenuation of redox fluctuations. This coincides with a marked reduction in stromatolite morphological variability (Fig. 8).

Increasing lines of evidence suggest that the Mesoproterozoic ocean was not a persistently anoxic system, but rather characterized by pronounced spatiotemporal heterogeneity in redox conditions. In shallow-water settings, intervals of transient oxygenation or the formation of localized “oxygen oases” may have occurred, whereas deeper environments remained largely anoxic or euxinic, forming complex redox-stratified water columns^6,54–57^. The widespread development of stromatolites recorded in Member II of the Longjiayuan formation provide new evidence for the coupling between microbial growth and localized oxygenation. These findings also contribute important insights into the diversity of oceanic redox regimes on a regional scale.

## Conclusions

This study conducts petrographic observations and geochemical analyses of siliceous-banded dolomites and stromatolites from the Longjiayuan Formation, located in the southern NCC. The main conclusions drawn from this study are summarized as follows:

1. Siliceous-banded dolomites are well developed in Member I of the Longjiayuan Formation, while Member II contains abundant stromatolites classified into stratiform, undulatory, domical, conical, and columnar types. The sedimentary environment of the Longjiayuan formation is primarily a tidal flat, with stromatolites developing in various sedimentary zones, including the intertidal and subtidal zones.
2. Ce anomalies in the Longjiayuan formation reveal fluctuating redox conditions, reflecting dynamic water column redox structures influenced by depositional depth and microbial activity.
3. The widespread development and morphological diversity of stromatolites in Member II reflect localized oxygen production by phototrophic microbial mats and enhanced nutrient availability, forming transient “oxygen oases” in shallow environments. These findings provide new insight into the coupling between microbial ecosystems and redox dynamics during the Proterozoic.

## Supplementary Information

Below is the link to the electronic supplementary material.

Supplementary Material 1

Supplementary Material 2

Supplementary Material 3

Supplementary Material 4

Supplementary Material 5

## Data Availability

The datasets generated during and analysed during the current study are available from the corresponding author on reasonable request.
