# Supplementary figures and images for: Stromatolites and pulsed oxygenation events in the Mesoproterozoic Longjiayuan formation of western Henan: evidence for life-environment co-evolution
Source: Sci Rep. 2025 Jul 29;15:27651. doi: 10.1038/s41598-025-13303-w (PMC12307726; doi:10.1038/s41598-025-13303-w)

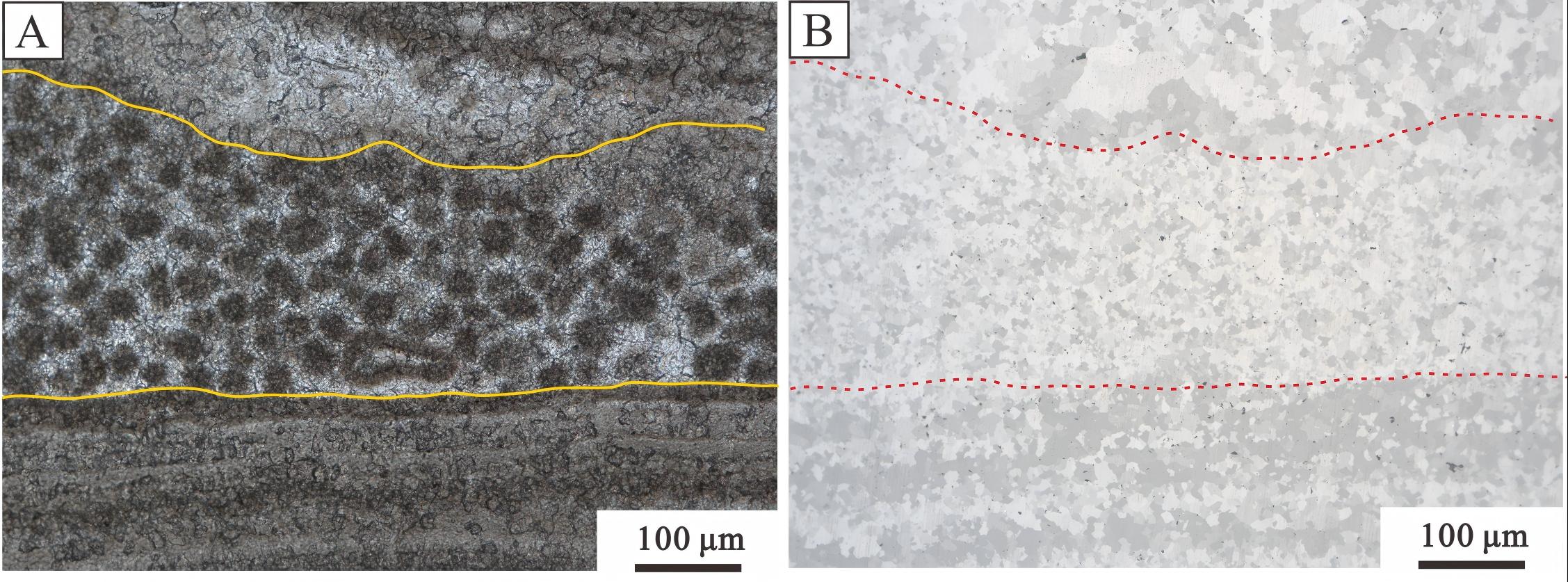

Supplement: Supplementary file 1 — Supplementary Material 1 [file 41598_2025_13303_MOESM1_ESM.png]

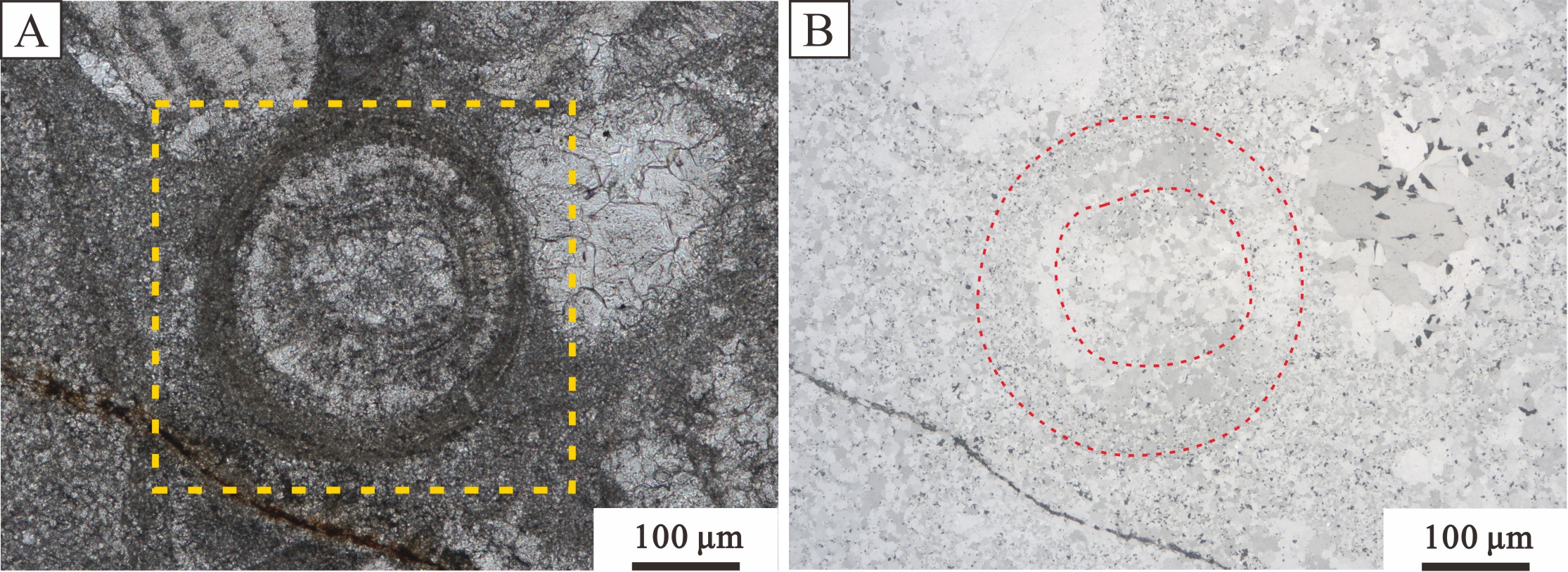

Supplement: Supplementary file 2 — Supplementary Material 2 [file 41598_2025_13303_MOESM2_ESM.png]

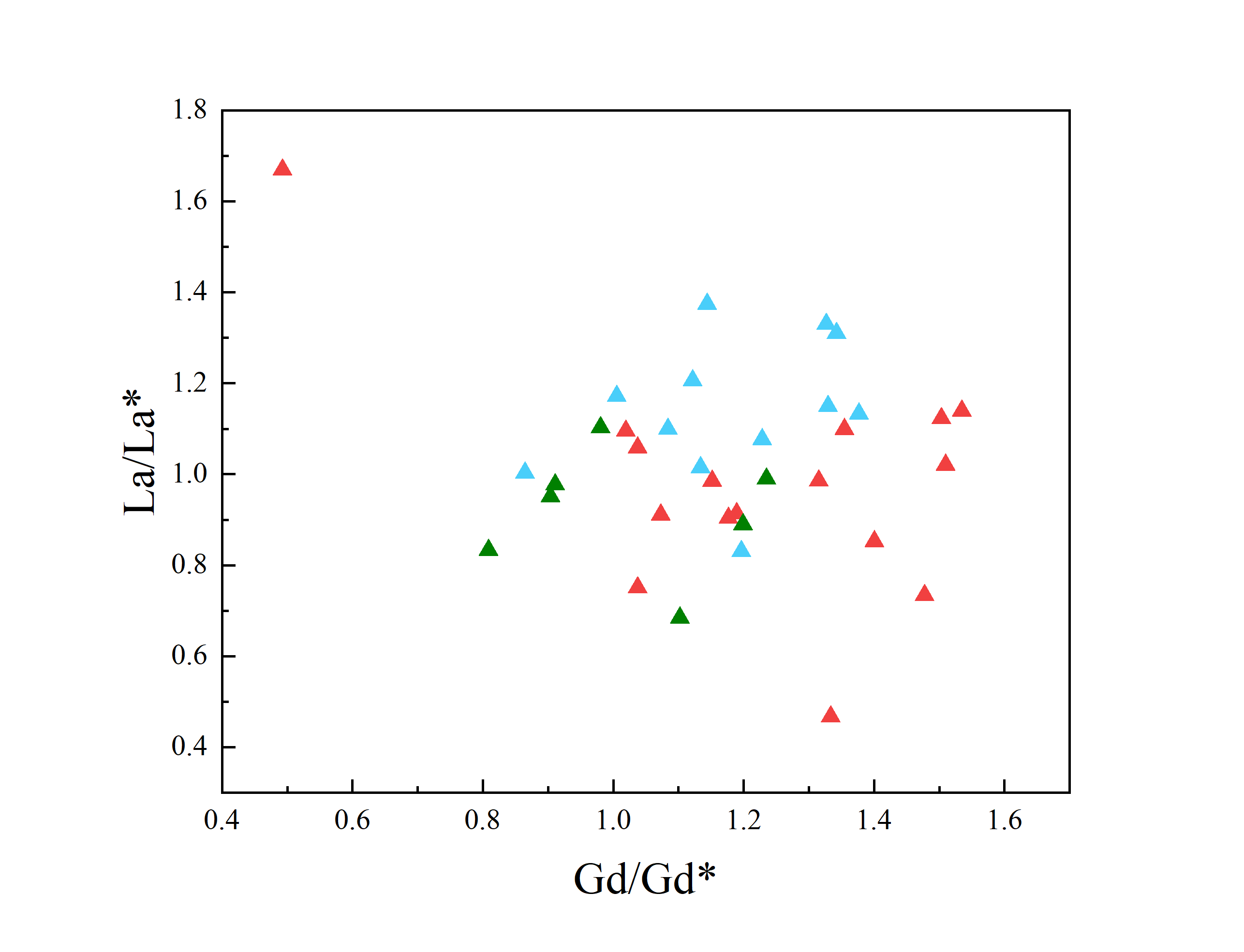

Supplement: Supplementary file 3 — Supplementary Material 3 [file 41598_2025_13303_MOESM3_ESM.png]
