# Supplementary material for: Stromatolites and pulsed oxygenation events in the Mesoproterozoic Longjiayuan formation of western Henan: evidence for life-environment co-evolution
Source: Sci Rep. 2025 Jul 29;15:27651. doi: 10.1038/s41598-025-13303-w (PMC12307726; doi:10.1038/s41598-025-13303-w)
Supplement: Supplementary file 4 — Supplementary Material 4 [file 41598_2025_13303_MOESM4_ESM.docx]

**Supplementary Figure Legends**


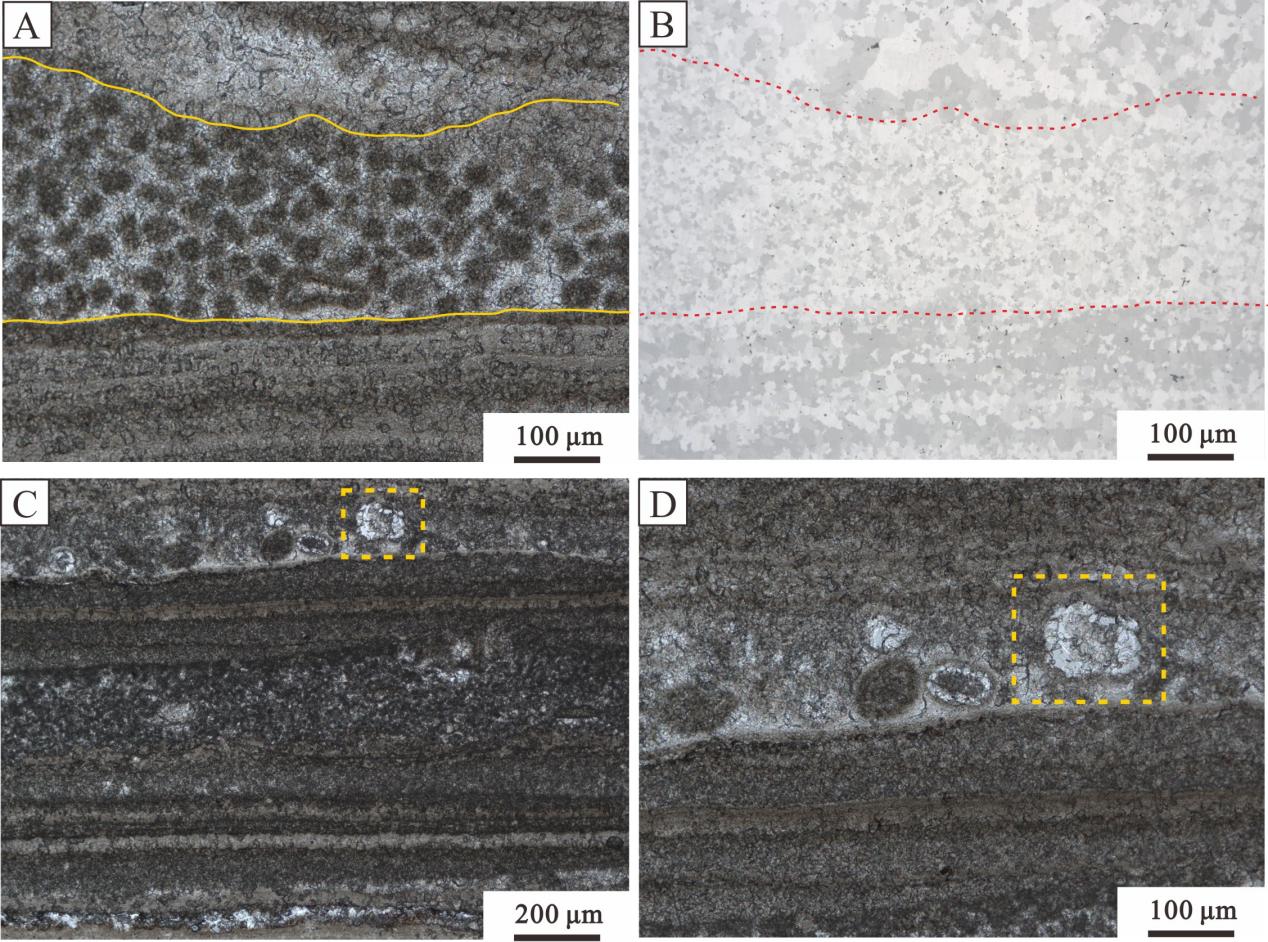
**Fig. S1. Microstructures of finely laminated stromatolites from Member II of the Longjiayuan Formation.**

**(A) Alternating light-dark laminae containing densely packed micritic spheroids (~5-10 μm in diameter), bounded by undulating microbial laminae (yellow lines); the spheroidal structures likely represent coccoid microbial aggregates.**

**(B) Reflected light photomicrograph of the same region as (A), showing diffuse boundaries of the micritic spheroids (red lines) within the stromatolitic matrix.**


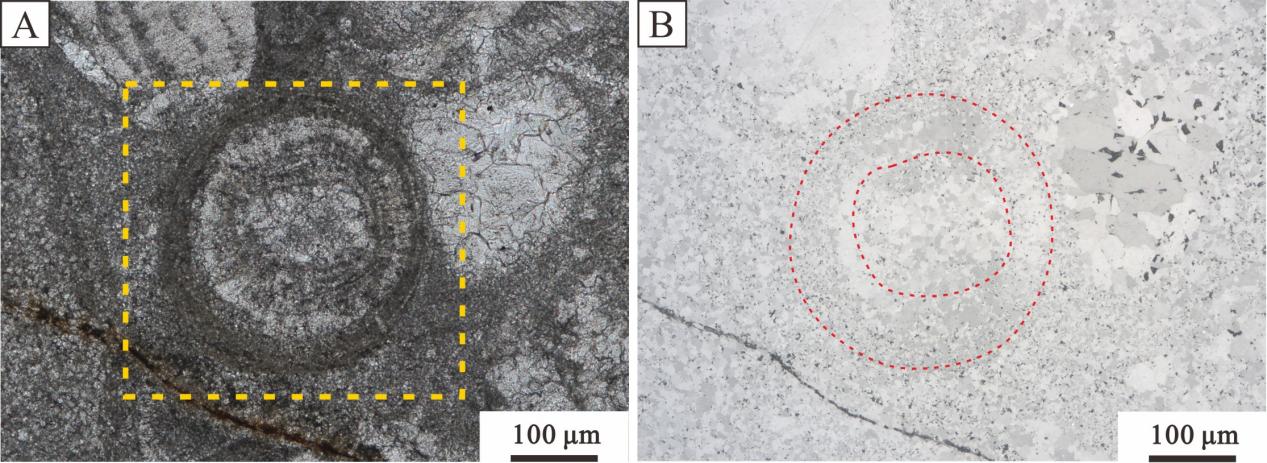


**Fig. S2. Photomicrographs showing concentric ooid laminae within stromatolitic dolomite of the Longjiayuan Formation.**

1. **Plane-polarized light (PPL) image showing well-preserved concentric lamination in the ooid cortex.**
2. **(B) Reflected light image enhancing the visibility of concentric structures in the ooid.**


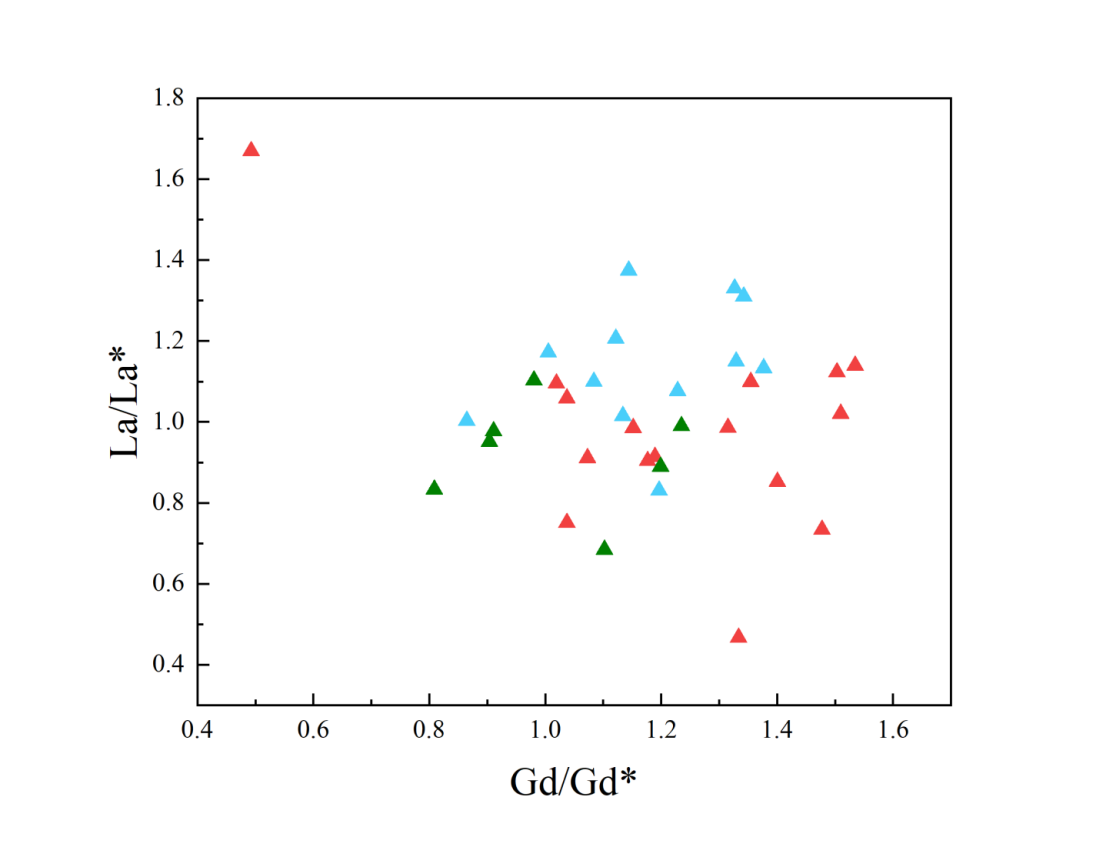


**Fig. S3. Cross plot of La/La* versus Gd/Gd* for carbonate samples from the Longjiayuan Formation. Most samples show near-unity to moderately positive anomalies, suggesting a discernible seawater REY signature. Red, blue, and green triangles represent samples from Members I, II, and III, respectively.**
