# Supplementary material for: Stromatolites and pulsed oxygenation events in the Mesoproterozoic Longjiayuan formation of western Henan: evidence for life-environment co-evolution
Source: Sci Rep. 2025 Jul 29;15:27651. doi: 10.1038/s41598-025-13303-w (PMC12307726; doi:10.1038/s41598-025-13303-w)
Supplement: Supplementary file 5 — Supplementary Material 5 [file 41598_2025_13303_MOESM5_ESM.pdf]

Permission to Publish Figure under CC BY 4.0 License

I, Shenghui Deng, confirm that I am the copyright holder of the original map figure published in the following article:

Tan, C., Lu, Y., Li, X., Song, H., Lv, D., Ma, X., Fan, R. and Deng, S. (2020). Carbon, oxygen and strontium isotopes of the Mesoproterozoic Jixian System (1.6-1.4 Ga) in the southern margin of the North China Craton and the geological implications. International Geology Review, 62(13-14), 1607-1623. https://doi.org/10.1080/00206814.2020.1818141

I hereby grant permission to Springer Nature Limited to publish a modified version of the above map figure in the manuscript entitled:

“Stromatolites and pulsed oxygenation events in the Mesoproterozoic Longjiayuan Formation of Western Henan: Evidence for life-environment co-evolution”

which is being submitted to Scientific Reports, under the terms of the Creative Commons Attribution 4.0 International (CC BY 4.0) license (https://creativecommons.org/licenses/by/4.0/). This permission covers all formats, including print and digital publication.

Name of copyright holder: Shenghui Deng

Signature: Shenghui Deng

Date: 2025.7.22
